# Facial Stimulation Induces Long-Term Potentiation of Mossy Fiber-Granule Cell Synaptic Transmission *via* GluN2A-Containing *N*-Methyl-D-Aspartate Receptor/Nitric Oxide Cascade in the Mouse Cerebellum

**DOI:** 10.3389/fncel.2022.863342

**Published:** 2022-03-30

**Authors:** Di Lu, Peng Wan, Yang Liu, Xian-Hua Jin, Chun-Ping Chu, Yan-Hua Bing, De-Lai Qiu

**Affiliations:** ^1^Department of Physiology and Pathophysiology, College of Medicine, Yanbian University, Yanji, China; ^2^Department of Ophthalmology, Affiliated Hospital of Yanbian University, Yanji, China; ^3^Department of Neurology, Affiliated Hospital of Yanbian University, Yanji, China; ^4^Department of Physiology, College of Basic Medicine, Jilin Medical University, Jilin, China

**Keywords:** cerebellum, mossy fiber-granule cell synaptic transmission, sensory stimulation, plasticity, NMDA receptor, nitric oxide, *in vivo* electrophysiological recording

## Abstract

Long-term synaptic plasticity in the cerebellar cortex is a possible mechanism for motor learning. Previous studies have demonstrated the induction of mossy fiber-granule cell (MF-GrC) synaptic plasticity under *in vitro* and *in vivo* conditions, but the mechanisms underlying sensory stimulation-evoked long-term synaptic plasticity of MF-GrC in living animals are unclear. In this study, we investigated the mechanism of long-term potentiation (LTP) of MF-GrC synaptic transmission in the cerebellum induced by train of facial stimulation at 20 Hz in urethane-anesthetized mice using electrophysiological recording, immunohistochemistry techniques, and pharmacological methods. Blockade of GABA_A_ receptor activity and repetitive facial stimulation at 20 Hz (240 pulses) induced an LTP of MF-GrC synapses in the mouse cerebellar cortical folium Crus II, accompanied with a decrease in paired-pulse ratio (N2/N1). The facial stimulation-induced MF-GrC LTP was abolished by either an *N*-methyl-D-aspartate (NMDA) receptor blocker, i.e., D-APV, or a specific GluNR2A subunit-containing NMDA receptor antagonist, PEAQX, but was not prevented by selective GluNR2B or GluNR2C/D subunit-containing NMDA receptor blockers. Application of GNE-0723, a selective and brain-penetrant-positive allosteric modulator of GluN2A subunit-containing NMDA receptors, produced an LTP of N1, accompanied with a decrease in N2/N1 ratio, and occluded the 20-Hz facial stimulation-induced MF-GrC LTP. Inhibition of nitric oxide synthesis (NOS) prevented the facial stimulation-induced MF-GrC LTP, while activation of NOS produced an LTP of N1, with a decrease in N2/N1 ratio, and occluded the 20-Hz facial stimulation-induced MF-GrC LTP. In addition, GluN2A-containing NMDA receptor immunoreactivity was observed in the mouse cerebellar granular layer. These results indicate that facial stimulation at 20 Hz induced LTP of MF-GrC synaptic transmission *via* the GluN2A-containing NMDA receptor/nitric oxide cascade in mice. The results suggest that the sensory stimulation-evoked LTP of MF-GrC synaptic transmission in the granular layer may play a critical role in cerebellar adaptation to native mossy fiber excitatory inputs and motor learning behavior in living animals.

## Introduction

Long-term synaptic plasticity is a type of modification of synaptic strength and is considered the synaptic mechanism of learning and memory in the brain (Bliss and Collingridge, 1993; Malenka and Nicoll, 1999). In cerebellar circuitry, long-term synaptic plasticity, including long-term potentiation (LTP) and long-term depression (LTD), has also been observed in the parallel fiber-Purkinje cell (PF-PC), parallel fiber-molecular layer interneuron (PF-MLI), molecular interneuron-Purkinje cell (MLI-PC), and mossy fiber-granule cell (MF-GrC) synapses, which has been considered a cellular mechanism for motor learning (Ito et al., 1982; Grasselli and Hansel, 2014).

The cerebellar granular layer mainly includes granule cells (GrCs) and Golgi cells. GrCs are the glutamatergic neurons in the cerebellar cortex and receive excitatory inputs from mossy fiber and relay them to PCs and MLIs through parallel fibers (Eccles et al., 1967). Cerebellar MF-GrC long-term synaptic plasticity has been well-demonstrated in acute cerebellar slices (D’Angelo et al., 1999; Rossi et al., 2002) and anesthetized animals (Roggeri et al., 2008). Using cerebellar slices, a type of MF-GrC LTP was induced by high-frequency stimulation (100 Hz, 10 pulses). The MF-GrC LTP was dependent on the activity of NMDA and metabotropic glutamate receptors (D’Angelo et al., 1999), especially GluN2A/GluN2C subunit-containing NMDA receptors (Rossi et al., 2002). Under *in vivo* conditions, it has been demonstrated that facial stimulation at 4 Hz induced LTD of local field potentials in the cerebellar granular layer under control conditions in urethane-anesthetized rats, but 4-Hz facial stimulation produced LTP when GABA_A_ receptors were blocked (Roggeri et al., 2008). However, no relevant plasticity was observed when both GABA_A_ and NMDA receptors were blocked (Roggeri et al., 2008). Both the 4-Hz facial stimulation-induced LTP and LTD in the rat cerebellar cortical Crus II were considered to be dependent on the local excitatory/inhibitory balance in the cerebellar granular layer under urethane-anesthetized conditions. LTD was induced in the presence of strong GABAergic inhibition, whereas LTP was induced in the absence of GABAergic inhibition (Mapelli and D’Angelo, 2007; Roggeri et al., 2008). However, the mechanism underlying the facial stimulation-induced LTP of MF-GrC synaptic transmission is still unclear.

Nitric oxide (NO) works as a retrograde signal that can facilitate or suppress plasticity through biphasic effects on presynaptic vesicle regulation, *S*-nitrosylation of synaptic proteins, and cyclic GMP generation (Reyes-Harde et al., 1999; Stanton et al., 2005; Ratnayaka et al., 2012; Selvakumar et al., 2013). Activation of NMDA receptors can induce calcium influx and activate nitric oxide synthase (NOS), which results in an increase in NO levels (Kim and Huganir, 1999; Szabadits et al., 2011). NO concentrations are likely to be modulated in an activity-dependent manner, such as the induction protocol of long-term synaptic plasticity in the central nervous system (Garthwaite, 2019). In the cerebellum, NO was implicated in presynaptically expressed plasticity at cerebellar PF-PC synapses (Qiu and Knöpfel, 2007; Chu et al., 2014) and the sensory stimulation-induced cerebellar MLI–PC LTD in a mouse model of chronic ethanol exposure (Li et al., 2019).

In this study, we investigated the possible mechanisms underlying 20-Hz facial stimulation-induced MF-GrC LTP in the mouse cerebellar granular layer in the absence of GABA_A_ receptor-mediated inhibitory inputs. Our results showed that facial stimulation at 20 Hz induced an LTP at the MF-GrC synapses *via* the GluN2A-containing receptor/NO cascade in mice. The results suggest that sensory stimulation-evoked MF-GrC LTP might play a critical role in cerebellar adaptation to native MF excitatory inputs and motor learning behavior in living animals.

## Materials and Methods

### Animals

A total of 76 adult (5-week-old) male HA/ICR mice were used in this study. The experimental procedures were approved by the Animal Care and Use Committee of Yanbian University and were in accordance with the animal welfare guidelines of the United States National Institutes of Health. The permit number is SYXK (Ji) 2011-006. All animals were housed under a 12-h light:12-h dark cycle with free access to food and water in a colony room under constant temperature (23 ± 1°C) and humidity (50 ± 5%).

### Anesthesia and Surgical Procedures

The anesthesia and surgical procedures have been described previously (Chu et al., 2011). The mice were anesthetized with urethane [1.3 g/kg body weight, intraperitoneal (i.p.)] and were tracheotomized to avoid respiratory obstruction. On a custom-made stereotaxic frame, soft tissue was retracted to gain access to the dorsal portion of the occipital bone. A watertight chamber was created, and a 1–1.5-mm craniotomy was drilled to expose the cerebellar surface corresponding to Crus II. The brain surface was constantly superfused with oxygenated artificial cerebrospinal fluid (ACSF: 125 mM NaCl, 3 m MKCl, 1 mM MgSO_4_, 2 mM CaCl_2_, 1 mM NaH_2_PO_4_, 25 mM NaHCO_3_, and 10 mM D-glucose) with a peristaltic pump (Gilson Minipulse 3; Villiers, Le Bel, France) at 0.4 ml/min. Rectal temperature was monitored and maintained at 37.0 ± 0.2°C using body temperature equipment.

### Electrophysiological Recording and Facial Stimulation

Local field potential recordings from granular layer were performed with an Axopatch 200B amplifier (Molecular Devices, Foster City, CA, United States) under current clamp conditions (*I* = 0). The potentials were acquired through a Digidata 1440 series analog-to-digital interface through a personal computer using Clampex 10.4 software. Recording electrodes were filled with ACSF and with resistances of 3–5 MΩ.

**FIGURE 1:**
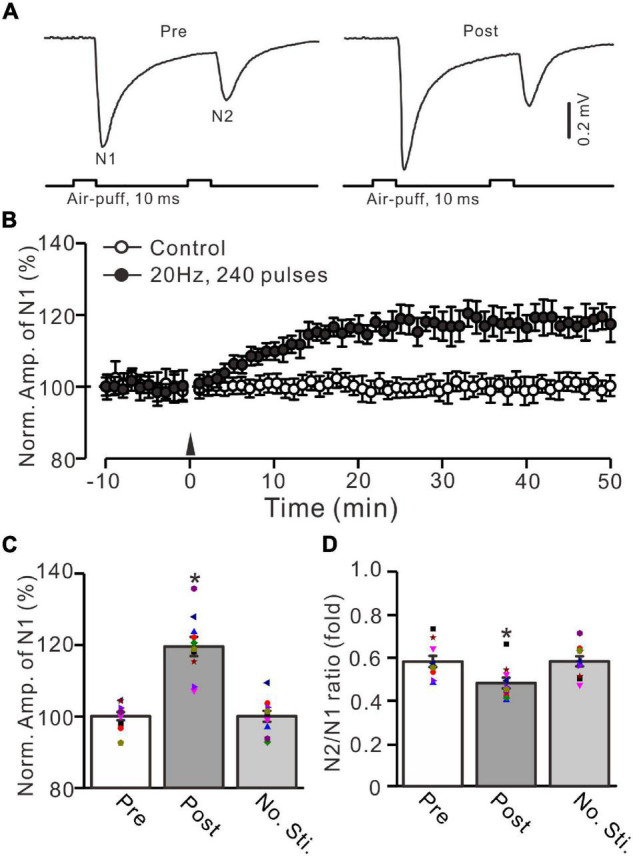
Facial stimulation at 20 Hz induced an MF-GrC LTP in the mouse cerebellar granular layer. **(A)** Upper: Representative extracellular recording traces showing a paired air-puff stimulation (10 ms, 60 psi, 50 ms interval) evoked MF-GrC synaptic responses before (pre) and after (post) the delivery of 20-Hz (240 pulses) air-puff stimulation. **(B)** Summary of data showing the time course of normalized amplitude of N1 in which the 20-Hz stimulation protocol (arrow) was delivered (filled circle) or not delivered (open circle) under control conditions. **(C)** Mean value (± SEM) with individual data showing the normalized amplitude before (pre) and after (post) the delivery of the 20-Hz stimulation protocol. **(D)** Mean value (± SEM) with individual data showing the normalized N2/N1 ratio before (pre) and after (post) the delivery of 20-Hz stimulation protocol. Note that the 20-Hz facial stimulation induced an LTP at MF-GrC synapse in the mouse cerebellum. *P < 0.05 vs. pre; *n* = 10 recordings from 10 mice.

Facial stimulation was performed by air-puff (10 ms, 60 psi) of the ipsilateral whisker pad through a 12-gauge stainless steel tube connected with a pressurized injection system (Picospritzer ^®^ III; Parker Hannifin Co., Pine Brook, NJ, United States). The air-puff stimulations were controlled by a personal computer and were synchronized with the electrophysiological recordings and delivered at 0.05 Hz *via* a Master 8 controller (A.M.P.I., Jerusalem, Israel) and Clampex10.3 software. For isolating MF-GrC synaptic transmission, picrotoxin (100 μM) was added to ACSF during all recordings in order to prevent GABA_A_ receptor-mediated inhibitory response. In the presence of GABA_A_ receptor antagonist, paired air-puff (10 ms, 50–60 psi, 50 ms interval) of the ipsilateral whisker pad evoked paired negative components N1 and N2 in granular layer of cerebellar cortical folium Crus II (Figure 1A). According to our previous studies (Bing et al., 2015; Ma et al., 2019; Li et al., 2021), N1 and N2 were identified as excitatory components, depending on the MF-GC synaptic transmission. For the induction of MF-GrC LTP, air-puff stimulation (10 ms, 240 pulses) were delivered at 20 Hz. The induction stimulation was delivered 10 min after a stable baseline recording.

### Immunohistochemistry and Imaging

Mice (*n* = 3) were deeply anesthetized with an i.p. injection of 7% chloral hydrate (5 ml/kg) and then transcardially perfused by 300 ml cold phosphate-buffered saline (PBS) at pH 7.4, followed by 300 ml of 4% paraformaldehyde (PFA, Sinopharm Chemical Reagent Co, China) PBS solution. Brain was postfixed in 4% PFA for 48 h at 4°C and washed with PBS 5 times at 10-min intervals. Separate the cerebellum from the brain with a razor blade. The cerebellum was exposed to 10% sucrose, 20% sucrose, and 30% sucrose in PBS for more than 6 h. After embedding in Tissue-Tek O.C.T. Compound (Beijing Zhong Shan Jin Qiao Biotechnology Co., China), the cerebellum was quickly frozen in −80°C refrigerator for 2 h. Then cerebellum was sectioned into 8 μm slices in the sagittal plane using a freezing microtome (CM1900, Leica, Germany) and attached the sections to microscope slides. Sections were rewarming at 25°C for 30 min and then fixed with 4°C precooled acetone for 20 min. Slices were stored at 4°C for immunohistochemical experiments.

Microscope slides were permeabilized with 0.3% Triton X-100 in PBS and then were blocked (10% donkey serum in PBS) and incubated in a primary antibody (rabbit anti-GluN2A, 1:50, abcam), followed by Alexa Fluor 555 donkey anti-rabbit (Life Tech, 1:1,000) and 4’,6-diamidino-2-phenylindole (DAPI, 1:1,000). The incubation time for the primary antibody was overnight at 4°C. For the secondary antibody and DAPI, the incubation time was 2 h at room temperature. Microscope slides with slices were then washed 3 times in PBS covered with a coverslip and sealed with nail polish. Fluorescence images were acquired by confocal laser scanning microscope (Nikon C2, Tokyo, Japan) (Mi et al., 2018; Wu et al., 2020). A large region image including Crus II was obtained on Nikon C2 laser confocal system with 10 × objective.

### Chemicals

The reagents, which included picrotoxin, D-(-)-2-amino-5-phosphonopentanoic acid (D-APV), N^G^-nitro-L-arginine (L-NNA), NOS inhibitor; *S*-nitroso-*N*-acetyl-D-penicillamine (SNAP), an NO donor were purchased from Sigma-Aldrich (Shanghai, China). PEAQX and TCN 237 were purchased from Tocris (Bristol, United Kingdom). DQP-1105 and GNE-0723 were purchased from Good Laboratory Practice Bioscience (GLPBIO; Shanghai, China). For experiments with L-NNA and SNAP, cerebellar surface was perfused with 200 μM of L-NNA and 500 μM of SNAP for 1 h before recordings were started, respectively. The drugs were dissolved in ACSF and applied directly onto the cerebellar surface by a peristaltic pump (0.5 ml/min).

### Data Analysis

The electrophysiological data were analyzed using Clampfit 10.6 software (Molecular Devices). The amplitude of the evoked field potential responses was maintained constant for an individual experiment before and after the delivery of 20-Hz facial stimulation. Values are expressed as the mean ± SEM. One-way ANOVA (*post hoc* multiple comparison; SPSS software) was used to determine the level of statistical significance among groups of data. A *P*-value below 0.05 was considered statistically significant.

## Results

### Facial Stimulation at 20 Hz Induced an *N*-Methyl-D-Aspartate Receptor-Dependent Long-Term Potentiation in Mouse Cerebellar Granular Layer

In the presence of GABA_A_ receptor antagonist picrotoxin (100 μM), a paired air-puff (10 ms, 50–60 psi, 50 ms interval) of the ipsilateral whisker pad evoked negative components N1 and N2 in the mouse cerebellar granular layer (Figure 1A). According to our previous studies (Ma et al., 2019; Li et al., 2021), N1 and N2 were identified as facial stimulation-evoked MF-GrC synaptic responses. The mean amplitudes of N1and N2 were 0.59 ± 0.09 mV (*n* = 10 experiments) and 0.34 ± 0.06 mV (*n* = 10 experiments), respectively (not shown). Based on the frequency properties of cerebellar GrC response to air-puff stimulation (Bing et al., 2015; Ma et al., 2019), we used facial stimulation at 20 Hz (240 pulses) to induce long-term synaptic plasticity in the cerebellar granular layer. This repetitive facial stimulation induced a persistent LTP of the MF-GrC synaptic response, which was expressed as an increase in the amplitude of N1 for over 50 min (Figure 1B). The normalized amplitude of N1 was increased to 119.5 ± 3.6% of baseline during 45–50 min after 20-Hz facial stimulation (*F* = 44.2, *P* < 0.0001, *n* = 10 experiments; Figure 1C). In contrast, the normalized amplitude of N1 was 100.7 ± 2.3% of baseline during 40–50 min under control conditions (*F* = 0.03, *P* = 0.85, *n* = 10 experiments: not shown). The N2/N1 ratio was 0.48 ± 0.024 during 45–50 min after 20-Hz facial stimulation, which was significantly lower than that of baseline (0.58 ± 0.026; *F* = 7.72, *P* = 0.012; *n* = 10 experiments; Figure 1D). In addition, the amplitude of N1 and the N2/N1 value exhibited no significant changes under control conditions (no 20-Hz facial stimulation: Figures 1B–D). These results indicate that facial stimulation at 20 Hz induces an MF-GrC LTP accompanied with a decrease in paired-pulse ratio in the mouse cerebellar granular layer.

**FIGURE 2:**
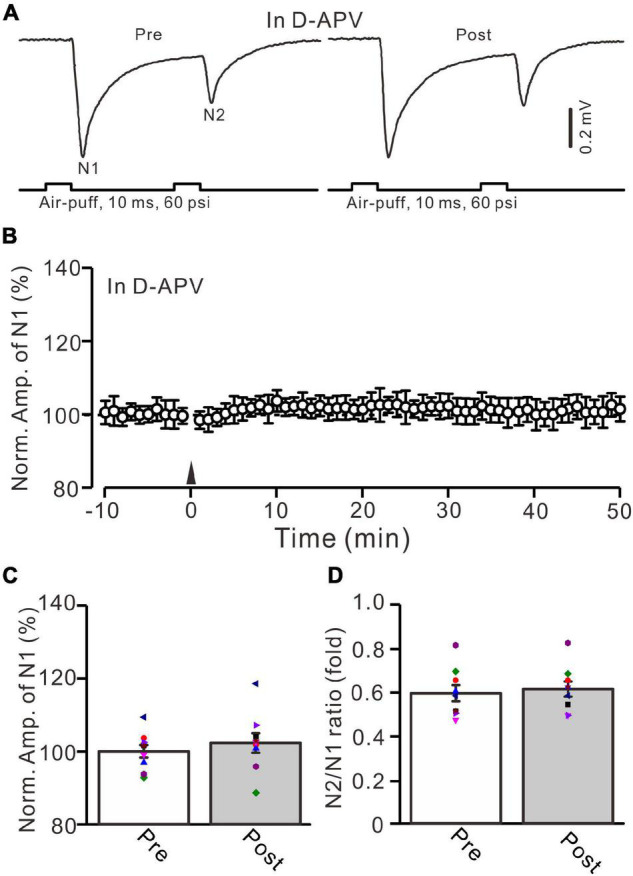
NMDA receptor blocker, D-APV, abolished the facial stimulation-induced MF-GrC LTP in the mouse cerebellar cortex. **(A)** Upper: Representative extracellular recording traces showing a paired air-puff stimulation (10 ms, 60 psi, 50 ms interval) evoked MF-GC synaptic responses before (pre) and after (post) the delivery of 20-Hz (240 pulses) air-puff stimulation in the presence of D-APV (250 μM). **(B)** Summary of data showing the time course of normalized amplitude of N1 in which the 20-Hz stimulation protocol (arrow) was delivered in the presence of D-APV. **(C)** Mean value (± SEM) with individual data showing the normalized amplitude before (pre) and after (post) the delivery of the 20-Hz stimulation protocol in the presence of D-APV. **(D)** Mean value (± SEM) with individual data showing the normalized N2/N1 ratio before (pre) and after (post) the delivery of the 20-Hz stimulation protocol in the presence of D-APV. Note that blockade NMDA receptor abolished the 20-Hz facial stimulation-induced MF-GrC LTP in the mouse cerebellum. *n* = 9 recordings from 9 mice.

NMDA receptors are expressed in the cerebellar granular layer and involved in MF-GrC synaptic transmission and long-term plasticity in mice (Roggeri et al., 2008; Zhang et al., 2020). We further examined whether the 20-Hz facial stimulation-induced MF-GrC LTP was mediated by NMDA receptors. In the presence of an NMDA receptor antagonist, i.e., D-APV (250 μM), facial stimulation at 20 Hz failed to induce MF-GrC LTP (Figure 2). The normalized amplitude of N1 was 102.6 ± 2.7% of baseline during 45–50 min after 20-Hz facial stimulation (*F* = 0.76, *P* = 0.58, *n* = 9 experiments; Figure 2C), and the N2/N1 ratio was 0.61 ± 0.034 during 45–50 min after 20-Hz facial stimulation, which was not significantly different than that of baseline (0.59 ± 0.037; *F* = 0.02, *P* = 0.91, *n* = 9 experiments; Figure 2D). These results indicate that the 20-Hz facial stimulation-induced MF-GrC LTP in the cerebellar granular layer is dependent on NMDA receptor activation.

### GluN2A-Containing *N*-Methyl-D-Aspartate Receptor Mediated the 20-Hz Facial Stimulation-Induced Cerebellar Mossy Fiber-Granule Cell Long-Term Potentiation

**FIGURE 3:**
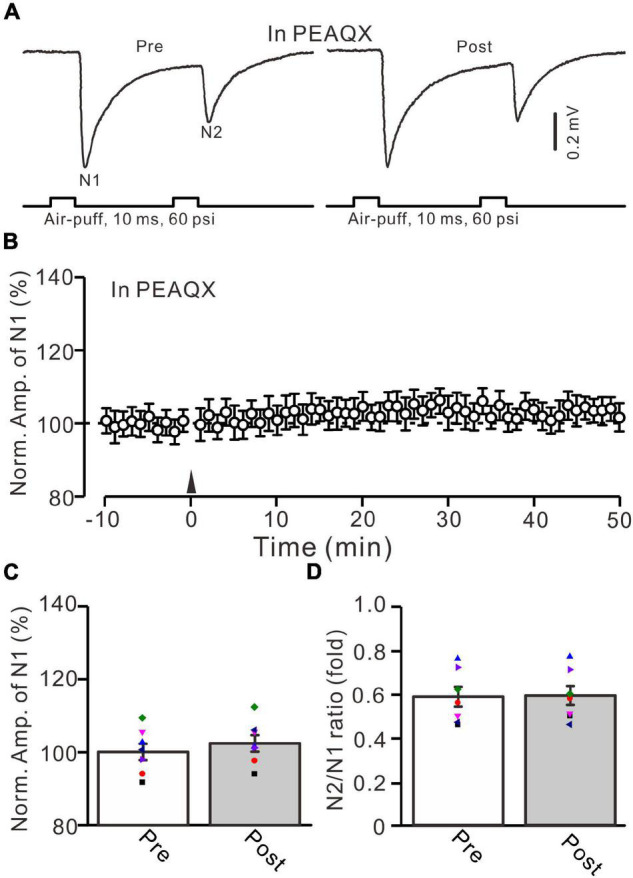
Blockade of GluN2A subunit-containing NMDA receptor blocker prevented the facial stimulation-induced MF-GrC LTP in the mouse cerebellar cortex. **(A)** Upper: Representative extracellular recording traces showing a paired air-puff stimulation (10 ms, 60 psi, 50 ms interval) evoked MF-GC synaptic responses before (pre) and after (post) the delivery of 20-Hz (240 pulses) air-puff stimulation in the presence of GluN2A subunit-containing NMDA receptor blocker, i.e., PEAQX (10 μM). **(B)** Summary of data showing the time course of normalized amplitude of N1 in which the 20-Hz stimulation protocol (arrow) was delivered in the presence of PEAQX. **(C)** Mean value (± SEM) with individual data showing the normalized amplitude before (pre) and after (post) the delivery of the 20-Hz stimulation protocol in the presence of PEAQX. **(D)** Mean value (± SEM) with individual data showing the normalized N2/N1 ratio before (pre) and after (post) the delivery of the 20-Hz stimulation protocol in the presence of PEAQX. Note that blockade GluN2A subunit-containing NMDA receptor abolished the 20-Hz facial stimulation-induced MF-GrC LTP in the mouse cerebellar granular layer. *n* = 7 recordings from 7 mice.

In the mouse cerebellar cortex, the expression of GluN2A, GluN2B, and GluN2C mRNA was detected in the cerebellar granular layer (Akazawa et al., 1994), and the GluN2A subunit was found abundantly at MF-GrC synapses in the adult mouse cerebellum (Yamada et al., 2001). We used a selective GluN2A subunit-containing NMDA receptor antagonist, i.e., PEAQX (10 μM), to examine whether the 20-Hz facial stimulation-induced MF-GrC LTP in the granular layer was dependent on the GluN2A subunit-containing NMDA receptor. In the presence of PEAQX, 20-Hz facial stimulation did not induce MF-GrC LTP in the mouse cerebellar granular layer (Figure 3). The normalized amplitude of N1 was 102.4 ± 2.3% of baseline during 45–50 min after 20-Hz facial stimulation (*F* = 0.81, *P* = 0.39, *n* = 7 experiments; Figure 3C). The N2/N1 ratio was 0.59 ± 0.042 during 45–50 min after 20-Hz facial stimulation, which was similar to that of baseline (0.58 ± 0.045; *F* = 0.012, *P* = 0.91, *n* = 7 experiments; Figure 3D). These results indicate that the 20-Hz facial stimulation-induced MF-GrC LTP in the cerebellar granular layer is dependent on the GluN2A subunit-containing NMDA receptor.

**FIGURE 4:**
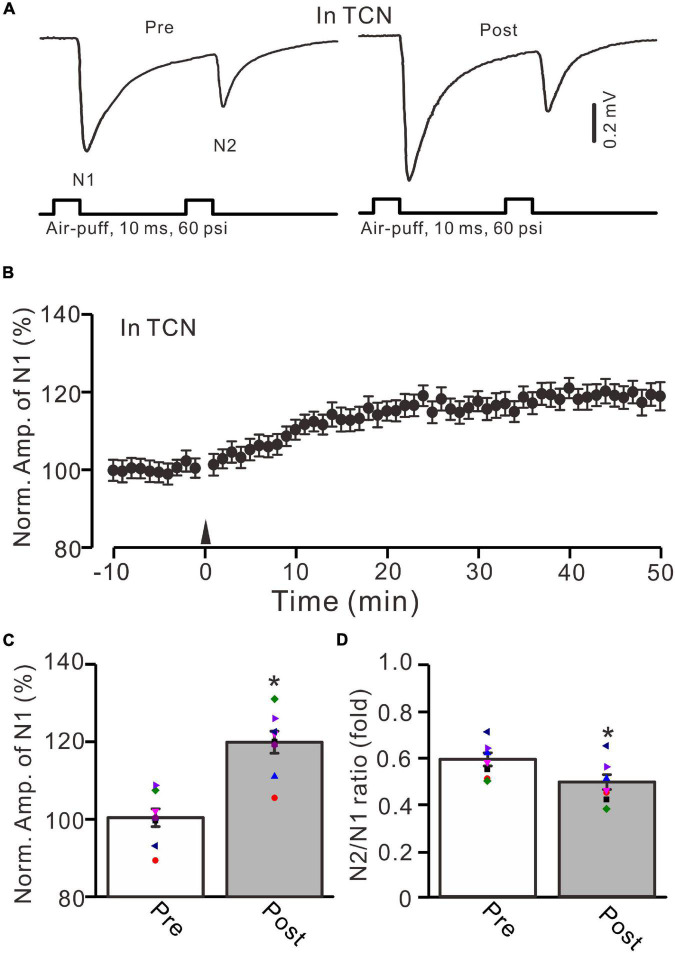
GluN2B subunit-containing NMDA receptor antagonist failed to prevent the facial stimulation-induced MF-GrC LTP in the mouse cerebellar granular layer. **(A)** Upper: Representative extracellular recording traces showing a paired air-puff stimulation (10 ms, 60 psi, 50 ms interval) evoked MF-GC synaptic responses before (pre) and after (post) the delivery of 20-Hz (240 pulses) air-puff stimulation in the presence of a GluN2B subunit-containing NMDA receptor antagonist, i.e., TCN (10 μM). **(B)** Summary of data showing the time course of normalized amplitude of N1 in which the 20-Hz stimulation protocol (arrow) was delivered in the presence of TCN. **(C)** Mean value (± SEM) with individual data showing the normalized amplitude before (pre) and after (post) the delivery of the 20-Hz stimulation protocol in the presence of TCN. **(D)** Mean value (± SEM) with individual data showing the normalized N2/N1 ratio before (pre) and after (post) the delivery of the 20-Hz stimuli protocol in the presence of TCN. Note that blockade GluN2B subunit-containing NMDA receptor failed to prevent the facial stimulation produced MF-GrC LTP in the mouse cerebellar granular layer. * *P* < 0.05 vs. pre; *n* = 8 recordings from 8 mice.

**FIGURE 5:**
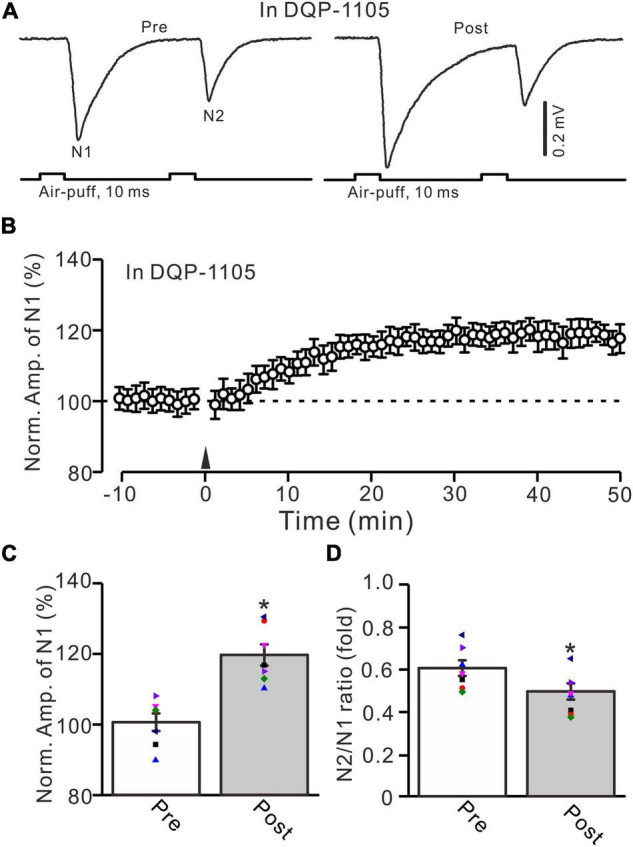
Blockade of GluN2C/D subunits-containing NMDA receptor did less effect on the facial stimulation-induced MF-GrC LTP in the mouse cerebellar granular layer. **(A)** Upper: Representative extracellular recording traces showing a paired air-puff stimulation (10 ms, 60 psi, 50 ms interval) evoked MF-GC synaptic responses before (pre) and after (post) the delivery of 20-Hz (240 pulses) air-puff stimulation in the presence of DQP-1105 (100 μM). **(B)** Summary of data showing the time course of normalized amplitude of N1 in which the 20-Hz stimuli protocol (arrow) was delivered in the presence of DQP-1105. **(C)** Mean value (± SEM) with individual data showing the normalized amplitude before (pre) and after (post) the delivery of the 20-Hz stimulation protocol in the presence of DQP-1105. **(D)** Mean value (± SEM) with individual data showing the normalized N2/N1 ratio before (pre) and after (post) the delivery of the 20-Hz stimulation protocol in the presence of DQP-1105. Note that blockade GluN2C/D subunits-containing NMDA receptor failed to abolish 20-Hz facial stimulation-induced MF-GrC LTP in the mouse cerebellar granular layer. * *P* < 0.05 vs. pre; *n* = 7 recordings from 7 mice.

Next, we employed a selective GluN2B subunit-containing NMDA receptor antagonist, i.e., TCN-237 (10 μM), to observe whether the GluN2B subunit-containing NMDA receptor is involved in 20-Hz facial stimulation-induced MF-GrC LTP in the cerebellar granular layer. In the presence of TCN-237 (10 μM), facial stimulation at 20 Hz induced MF-GrC LTP in the mouse cerebellar granular layer (Figures 4A,B). The normalized amplitude of N1 was 119.9 ± 2.9% of baseline during 45–50 min after 20-Hz facial stimulation (*F* = 31.2, *P* = 0.0003, *n* = 8 experiments; Figure 4C), and the N2/N1 ratio was 0.58 ± 0.042 during 40–50 min after 20-Hz facial stimulation, which was significantly lower than that of baseline (0.50 ± 0.04; *F* = 6.58, *P* = 0.04, *n* = 8 experiments; Figure 4D). These results indicate that the 20-Hz facial stimulation-induced MF-GrC LTP in the cerebellar granular layer is independent of the GluN2B subunit-containing NMDA receptor. Moreover, we used a selective GluN2C/D subunit-containing NMDA receptor antagonist, i.e., DQP-1105 (100 μM), to observe whether the GluN2C/D subunit-containing NMDA receptor is involved in facial stimulation-induced MF-GrC LTP in the mouse cerebellar granular layer. Administration of DQP-1105 (100 μM) did not abolish the 20-Hz facial stimulation-induced LTP of the excitatory response in the mouse cerebellar granular layer (Figures 5A,B). The normalized amplitude of N1 was 118.8 ± 3.1% of baseline during 45–50 min after 20-Hz facial stimulation (*F* = 35.1, *P* = 0.0002, *n* = 7 experiments; Figure 5C), and the N2/N1 ratio was 0.49 ± 0.031 during 45–50 min after 20-Hz facial stimulation, which was significantly lower than that of baseline (0.59 ± 0.028; *F* = 6.63, *P* = 0.032, *n* = 7 experiments; Figure 5D). These results indicate that the 20-Hz facial stimulation-induced MF-GrC LTP in the mouse cerebellar granular layer is independent of the GluN2C/D subunit-containing NMDA receptor.

**FIGURE 6:**
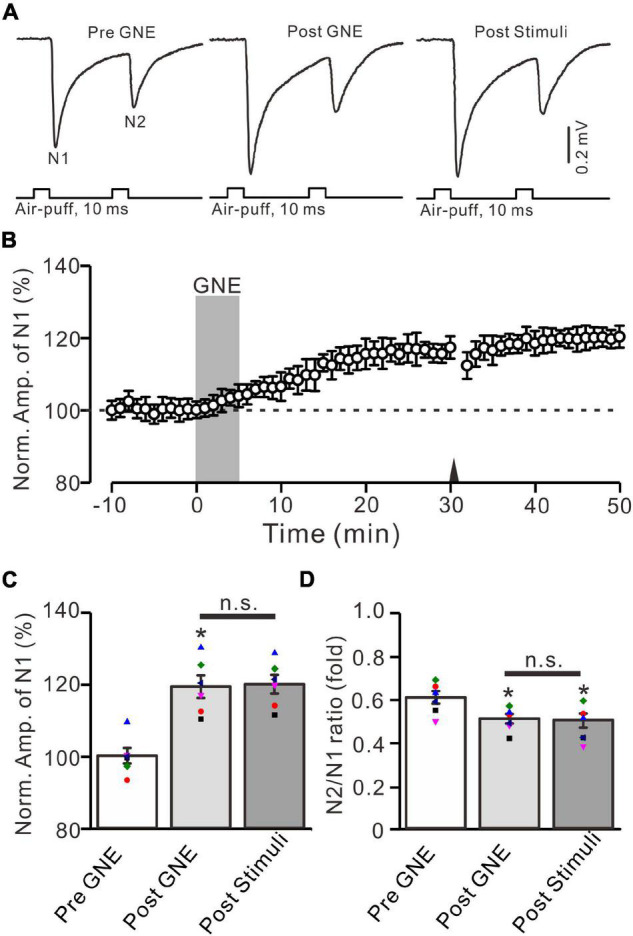
A selective positive allosteric modulator of GluN2A subunit-containing NMDA receptor, i.e., GNE-0723, produced MF-GrC LTP and occluded the 20-Hz stimulation protocol-induced LTP of MF-GrC synaptic transmission. **(A)** Upper: Representative extracellular recording traces showing a paired air-puff stimulation (10 ms, 60 psi, 50 ms interval) evoked MF-GC synaptic responses before (pre-GNE), after (post-GNE) application of GNE-0723 (10 μM; gray), and after delivery of the stimulation train (poststimuli). **(B)** Summary of data showing the time course of normalized amplitude of N1 before and after application of GNE-0723 (10 μM; gray) and after delivery of the stimulation train (arrow). **(C)** Mean value (± SEM) with individual data showing the normalized amplitude before (pre-GNE) and after (post-GNE) application of NMDA and after the stimulation train (poststimuli). **(D)** Mean value (± SEM) with individual data showing the normalized N2/N1 ratio before (pre-GNE) and after (post-GNE) application of GNE-0723 and after delivery of the stimulation train (poststimuli). Note that perfusion of GNE-0723 receptor produced an MF-GrC LTP and occluded the 20-Hz facial stimuli-induced LTP of MF-GrC synaptic transmission. * *P* < 0.05 vs. pre; *n* = 6 recordings from 6 mice.

The present results show that the 20-Hz facial stimulation-induced MF-GrC LTP in the mouse cerebellar granular layer was abolished by GluN2A subunit-containing NMDA receptor antagonist, but not by GluN2B and GluN2C/D subunit-containing NMDA receptor antagonists, suggesting that the MF-GrC LTP in the cerebellar granular layer was induced by activation of the GluN2A subunit-containing NMDA receptor. We next observed whether pharmacological activation of GluN2A subunit-containing NMDA receptors could induce MF-GrC LTP in the mouse cerebellar granular layer. To activate GluN2A subunit-containing NMDA receptors, we applied a selective and brain-penetrant-positive allosteric modulator of GluN2A subunit-containing NMDA receptors, i.e., GNE-0723 (10 μM; Villemure et al., 2016). The application of GNE-0723 for 5 min produced a significant increase in amplitude of N1 for over 30 min (Figures 6A,B). The normalized amplitude of N1 was 119.3 ± 3.1% of baseline during 25–30 min after application of GNE-0723 (*F* = 25.7, *P* = 0.0005, *n* = 6 experiments; Figure 6C), and the N2/N1 ratio was 0.51 ± 0.021 during 25–30 min after application of GNE-0723, which was significantly lower than that of baseline (0.60 ± 0.029; *F* = 7.38, *P* = 0.021, *n* = 6 experiments; Figure 6D). We further applied 20-Hz facial stimulation at 31 min after the application of GNE-0723 to observe whether 20-Hz facial stimulation could produce additional potentiation of MF-GrC synaptic transmission. The results showed that delivery of 20-Hz facial stimulation failed to induce additional potentiation of N1 amplitude (Figures 6A,B). The normalized amplitude of N1 was 119.9 ± 2.6% of baseline during 15–20 min after 20-Hz facial stimulation, which was not significantly different than that during 25–30 min after application of GNE-0723 (*F* = 0.023, *P* = 0.86, *n* = 6 experiments; Figure 6C). The N2/N1 ratio was 0.49 ± 0.03 during 15–20 min after 20-Hz facial stimulation, which was not significantly different than that during 25–30 min after application of GNE-0723 (*F* = 0.021, *P* = 0.87, *n* = 6 experiments; Figure 6D). These results indicate that pharmacological activation of GluN2A subunit-containing NMDA receptors produces LTP and occludes 20-Hz facial stimulation-induced MF-GrC LTP in the mouse cerebellar granular layer.

### The 20-Hz Facial Stimulation-Induced Long-Term Potentiation of Excitatory Response in Granular Layer Was Mediated by Nitric Oxide Cascade

**FIGURE 7:**
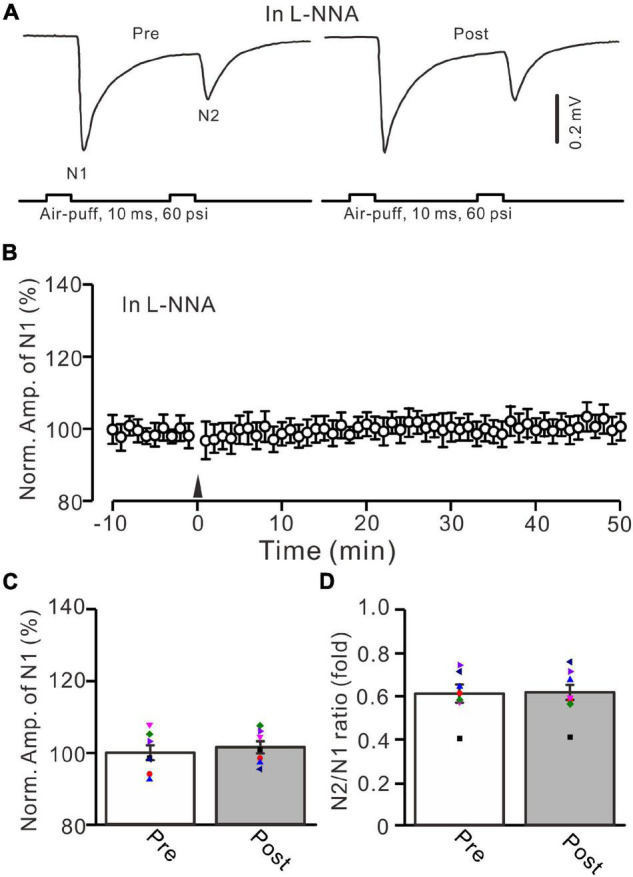
Inhibition of nitric oxide synthase abolished the facial stimulation-induced MF-GrC LTP in the mouse cerebellar cortex. **(A)** Upper: Representative extracellular recording traces showing a paired air-puff stimulation (10 ms, 60 psi, 50 ms interval) evoked MF-GrC synaptic responses before (pre) and after (post) delivery of 20-Hz (240 pulses) air-puff stimuli in the presence of nitric oxide synthase inhibitor, i.e., L-NNA (200 μM). **(B)** Summary of data showing the time course of normalized amplitude of N1 in which the 20-Hz stimuli protocol (arrow) was delivered in the presence of L-NNA. **(C)** Mean value (± SEM) with individual data showing the normalized amplitude before (pre) and after (post) the delivery of the 20-Hz stimulation protocol in the presence of L-NNA. **(D)** Mean value (± SEM) with individual data showing the normalized N2/N1 ratio before (pre) and after (post) the delivery of the 20-Hz stimulation protocol in the presence of L-NNA. Note that inhibiting nitric oxide synthase abolished the 20-Hz facial stimulation-induced MF-GrC LTP. *n* = 7 recordings from 7 mice.

In cerebellar cortical slices, high-frequency MF stimulation-induced LTP at MF-GrC synapses was dependent on the activation of NOS (Maffei et al., 2003), suggesting that NOS might be involved in the 20-Hz facial stimulation-induced LTP of excitatory responses in the mouse cerebellar granular layer. We further examined the 20-Hz facial stimulation-induced MF-GrC LTP in the presence of an NOS inhibitor, i.e., l-NNA (200 μM). After perfusion of l-NNA for 1 h, facial stimulation failed to induce MF-GrC LTP in the mouse cerebellar granular layer (Figures 7A,B). The mean amplitude of N1 was 101.4 ± 1.7% of baseline (100 ± 2.1%; *F* = 0.045, *P* = 0.57; *n* = 7; Figure 7C) at 45–50 min after delivery of 20-Hz facial stimulation. The mean value of N2/N1 ratio was 0.61 ± 0.03 during 45–50 min after 20-Hz facial stimulation, which was not significantly different than that of baseline (*F* = 0.019, *P* = 0.91, *n* = 7 experiments; Figure 7D). These results indicate that facial stimulation-induced MF-GrC LTP in the mouse cerebellar granular layer is also dependent on NOS activation.

**FIGURE 8:**
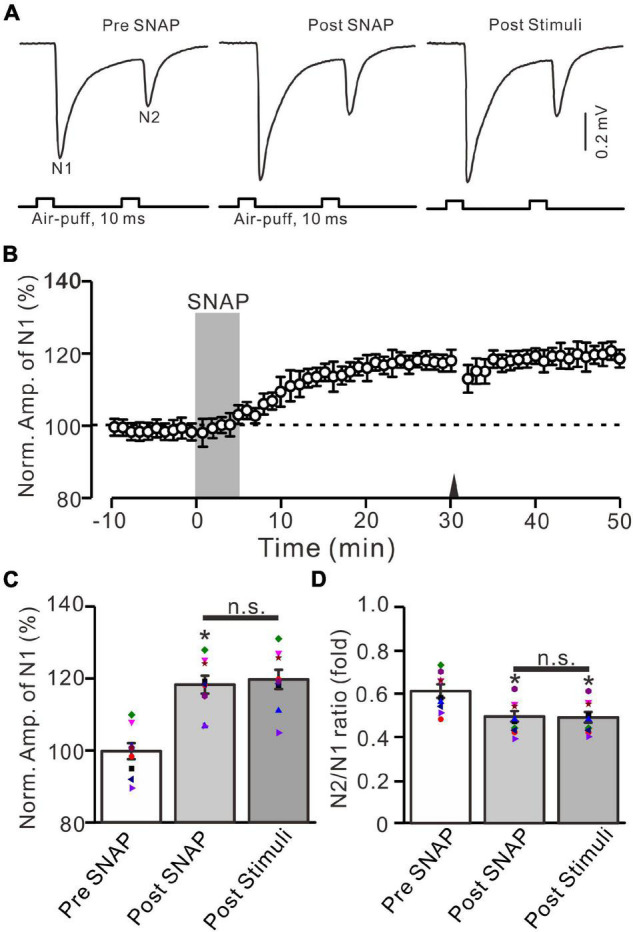
Application of NO donor produced MF-GrC LTP, which prevented the 20-Hz stimulation protocol-induced LTP of MF-GrC synaptic transmission. **(A)** Upper: Representative extracellular recording traces showing a paired air-puff stimulation (10 ms, 60 psi, 50 ms interval) evoked MF-GC synaptic responses before (pre-SNAP) and after (post-SNAP) application of NO donor, i.e., SNAP (500 μM; gray), and after delivery of the stimulation train (poststimuli). **(B)** Summary of data showing the time course of normalized amplitude of N1 before and after application of SNAP (100 μM; gray) and after delivery of the stimulation train (arrow). **(C)** Mean value (± SEM) with individual data showing the normalized amplitude before (pre-SNAP) and after (post-SNAP) application of SNAP and after the stimulation train (poststimuli). **(D)** Mean value (± SEM) with individual data showing the normalized N2/N1 ratio before (pre-SNAP) and after (post-SNAP) application of SNAP and after delivery of the stimulation train (poststimuli). Note that perfusion of NO donor produced an LTP of MF-GrC synaptic transmission and prevented the 20-Hz facial stimuli-induced MF-GrC LTP. * *P* < 0.05 vs. pre; *n* = 9 recordings from 9 mice.

In addition, the application of SNAP (500 μM) for 5 min produced a significant increase in the amplitude of N1 for over 30 min (Figures 8A,B). The normalized amplitude of N1 was 118.1 ± 2.5% of baseline during 25–30 min after the application of SNAP (*F* = 25.9, *P* = 0.0005, *n* = 9 experiments; Figure 8C), and the N2/N1 ratio was 0.49 ± 0.025 during 25–30 min after the application of SNAP (F = 8.1, P = 0.012 vs. baseline, *n* = 9 experiments; Figure 8D). Notably, the application of 20-Hz facial stimulation at 31 min failed to produce additional LTP of MF-GrC synaptic transmission (Figures 8A,B). The normalized amplitude of N1 was 119.7 ± 2.7% of baseline during 15–20 min after 20-Hz facial stimulation, which was not significantly different than that during 25–30 min after SNAP application (*F* = 0.024, *P* = 0.79, *n* = 9 experiments; Figure 8C). The mean N2/N1 ratio was 0.48 ± 0.024 during 15–20 min after 20-Hz facial stimulation, which was not significantly different than that during 25–30 min after the application of SNAP (*F* = 0.04, *P* = 0.85, *n* = 9 experiments; Figure 8D). These results indicate that pharmacological activation of NOS could induce MF-GrC LTP and occlude 20-Hz facial stimulation-induced MF-GrC LTP in the mouse cerebellar granular layer.

**FIGURE 9:**
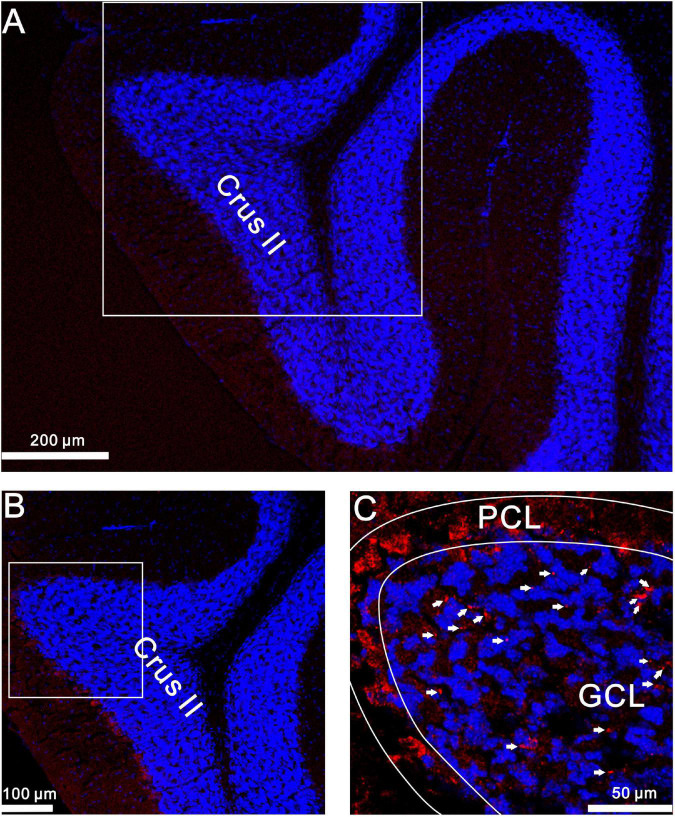
GluN2A immunoreactivity was expressed in GrCs of the mouse cerebellar cortical lobule Crus II. **(A)** A digital micrograph shows the confocal image of DAPI (blue) in the mouse cerebellar lobule Crus II. DAPI is a blue nucleic acid dye that preferentially dyes the dsDNA of cells. **(B)** Higher magnifications of the boxed area in **(A)**. **(C)** Higher magnifications of the boxed area in **(B)** showing GluN2A subunit-containing NMDA receptor immunoreactivity expressed in GL (red; arrows). ML, molecular layer; PCL, Purkinje cell layer; GL, granular layer.

Finally, we used confocal laser scanning microscopy to observe whether GluN2A-containing NMDA receptors were expressed in the cerebellar granular layer. Our results showed that GluN2A subunit-containing NMDA receptor immunoreactivity was present in the mouse granular layer (Figure 9). Together, our results suggest that the 20-Hz facial stimulation-induced MF-GrC LTP in the mouse cerebellar granular layer occurs through the GluN2A-containing NMDA receptor/NO cascade in mice.

## Discussion

In this study, we demonstrated that facial stimulation at 20 Hz induced MF-GrC LTP accompanied with a decrease in paired-pulse ratio in the mouse cerebellar granular layer. The facial stimulation-induced MF-GrC LTP was abolished by either an NMDA receptor blocker or a GluR2A subunit-containing NMDA receptor antagonist, but was not sensitive to GluNR2B or GluNR2C/D subunit-containing NMDA receptor blockers. Notably, the application of a selective positive allosteric modulator of the GluN2A subunit-containing NMDA receptor or an NO donor not only mimicked but also occluded the facial stimulation-evoked MF-GrC LTP in the mouse cerebellar GrC layer. These results indicate that facial stimulation at 20 Hz induced LTP of MF-GrC excitatory synaptic transmission *via* a GluN2A receptor/NO cascade in mice.

### Repeated Facial Stimulation Induced an *N*-Methyl-D-Aspartate Receptor-Dependent Long-Term Potentiation of Mossy Fiber-Granule Cell Synaptic Transmission in the Mouse Cerebellar Granular Layer

Long-term synaptic plasticity at MF-GrC, including LTD and LTP, has been previously investigated in acute cerebellar slices (D’Angelo et al., 1999; Sola et al., 2004; Gall et al., 2005; D’Errico et al., 2009; Gao et al., 2012). The expression of MF-GrC LTP or LTD has been proposed to be dependent on the balance of local excitation and inhibition, especially the amount of MF excitatory input and the intensity of Golgi cell-mediated GABAergic inhibition (Mapelli and D’Angelo, 2007). In addition, the induction of MF-GrC LTP or LTD is strongly regulated by the plasticity of the Golgi cell loop (Garrido et al., 2013). Under *in vivo* conditions, the induction pattern, pharmacology, and kinetics of MF-GrC synaptic plasticity are compatible with *in vitro* experiments (Mapelli and D’Angelo, 2007). In the cerebellar cortex, sensory stimulation evokes field potential responses in the granular layer *via* MF activation, which is supposed to originate from spike firing currents in clusters of GrCs. The facial stimulation-evoked response in the cerebellar granular layer can be persistently modified by patterned induction stimulation and produces long-term synaptic plasticity at MF-GrC synapses (Diwakar et al., 2011). Facial stimulation at 4 Hz induces LTD at the granular layer in the presence of Golgi cell-mediated GABAergic inhibition and induces LTP in the absence of GABAergic inhibition (Roggeri et al., 2008), suggesting that GABAergic inhibition plays a critical role during the induction of plasticity in the cerebellar granular layer. Consistent with previous studies (Mapelli and D’Angelo, 2007; Roggeri et al., 2008), our results showed that the 20-Hz facial stimulation-induced LTP in the mouse cerebellar granular layer coincided with a decrease in paired-pulse ratio, suggesting that the facial stimulation-induced MF-GrC LTP is involved in a change in neurotransmitter release from MFs (Qiu and Knöpfel, 2007). In addition, GrCs express high-frequency properties and can produce high-fidelity responses to facial stimulation higher than 30 Hz (Bing et al., 2015). High-frequency (100 Hz) stimulation of MFs paired with GrC membrane depolarization could induce an NMDA receptor-dependent MF-GrC LTP in cerebellar slices (D’Angelo et al., 1999). Therefore, we employed facial stimulation at 20 Hz (240 pulses), but not 4 Hz (Roggeri et al., 2008), for induction of MF-GrC LTP in the mouse cerebellar cortex.

NMDA receptors are expressed in the cerebellar granular layer and are related to MF-GrC synaptic transmission (D’Angelo et al., 1993, 1995; Kadotani et al., 1996) and long-term synaptic plasticity (D’Angelo et al., 1999; Roggeri et al., 2008). Consistent with previous studies (D’Angelo et al., 1999; Roggeri et al., 2008), the present results show that the 20-Hz facial stimulation-induced LTP of excitatory components was abolished by an NMDA receptor antagonist. A previous study showed that GluN2A, GluN2B, and GluN2C mRNA were expressed in mouse cerebellar GrCs (Akazawa et al., 1994). Our results showed that the 20-Hz facial stimulation-induced LTP of MF-GrC synaptic transmission was prevented by a specific GluN2A subunit-containing NMDA receptor antagonist. Notably, activation of GluN2A subunit-containing NMDA receptors produced LTP and occluded 20-Hz facial stimulation-induced MF-GrC LTP in the mouse cerebellum. Moreover, facial stimulation-induced MF-GrC LTP persisted in the presence of GluN2B subunit-containing NMDA receptor blocker, suggesting that the 20-Hz facial stimulation-induced LTP of MF-GrC synaptic transmission was not dependent on the GluN2B subunit-containing NMDA receptor. It has been reported that the GluN2C subunit-containing NMDA receptor is preferentially incorporated into triheteromeric GluN1/GluN2A/GluN2C receptors in cerebellar GrCs. The triheteromeric GluN1/GluN2A/GluN2C receptors express single-channel properties that cannot be predicted from the composite subunits (Bhattacharya et al., 2018). However, the application of GluN2C/D subunit-containing NMDA receptor blocker failed to prevent facial stimulation-induced MF-GrC LTP in the mouse cerebellum, suggesting that the diheteromeric GluN1/GluN2A receptors, but not the triheteromeric GluN1/GluN2A/GluN2 receptors, may have a dominant role in the 20-Hz facial stimulation-induced MF-GrC LTP in mice. The present results are supported by previous studies (Akazawa et al., 1994; Yamada et al., 2001; Rossi et al., 2002). First, GluN2A subunit-containing NMDA receptors were found abundantly at the MF-GrC synapses (Akazawa et al., 1994; Yamada et al., 2001). Furthermore, GluN2A subunit-containing NMDA receptors regulate synaptic activation and function by enhancing channel open probability, which plays a critical role in LTP induction (Rossi et al., 2002). Moreover, our results showed that GluN2A subtype NMDA receptor immunoreactivity was present in the mouse cerebellar granular layer.

### Possible Mechanisms of the 20-Hz Facial Stimulation-Induced Long-Term Potentiation of Excitatory Components in the Cerebellar Granular Layer

The mechanisms of NMDA-dependent long-term synaptic plasticity have been well-demonstrated (Rossi et al., 2002). The activation of GluN2 subtype NMDA receptors may induce calcium influx through NMDA receptor channels and activate postsynaptic density-associated calcium-sensitive enzymes, such as NOS and protein kinase (Kim and Huganir, 1999). Our results showed that inhibition of NOS abolished the facial stimulation-induced LTP of MF-GrC synaptic transmission, suggesting that facial stimulation-induced LTP of the excitatory response in the granular layer is dependent on NOS activation. Notably, the application of NO donor induced LTP of MF-GrC synaptic transmission and occluded the facial stimulation to induce additional MF-GrC LTP in the mouse cerebellum. These results indicate that facial stimulation-induced LTP of MF-GrC synaptic transmission is dependent on GluN2A subunit-containing NMDA receptors and NOS activation. NMDA receptor channels have a high permeability to Ca^2+^, and activation of these channels produces an increase in intracellular calcium concentration and Ca^2+^-calmodulin activation (Garthwaite, 2019), resulting in activation of NOS in the cerebellar cortical granular layer (Bredt and Snyder, 1990; Bredt et al., 1991). NO is a retrograde messenger that acts on presynaptic terminals when postsynaptic NMDA receptors are activated. NO concentrations are likely to be operated in an activity-dependent manner, such as the induction of synaptic plasticity in the central nervous system (Garthwaite, 2019). Thus, NO could be generated by cerebellar GrCs during the 20-Hz facial stimulation *via* activation of the GluN2A subunit-containing NMDA receptor, resulting in LTP of MF-GrC synaptic transmission in the mouse cerebellum (Garthwaite et al., 1988).

### Physiological Significant of the Facial Stimulation-Induced Long-Term Potentiation in the Cerebellar Granular Layer

Long-term synaptic plasticity is a type of modification of synaptic strength that is considered a synaptic mechanism of learning and memory in the brain (Bliss and Collingridge, 1993; Malenka and Nicoll, 1999). Sensory information evokes MF-GrC excitatory synaptic transmission and long-term synaptic plasticity at the granular layer, which may play critical roles in motor coordination and motor learning (Roggeri et al., 2008; D’Angelo and De Zeeuw, 2009; Solinas et al., 2010). Our present results provide evidence for MF-GrC plasticity in contrast with Marr’s (1969) assumption that the MF-GrC synapse is not modifiable, extending the number of possible motor learning sites in the cerebellar cortex (Linden, 1997; D’Angelo et al., 1999). The MF-GrC LTP represents a great potential for cerebellar learning and memory because there are largest number GrCs and MF-GrC synapses to store information in cerebellar neuronal networks (D’Angelo et al., 1999). In addition, MF-GrC LTP may regulate the spatiotemporal patterns transmitted to PCs, which may determine GrC spike firing within proper time windows (Nieus et al., 2006; Mapelli and D’Angelo, 2007) and serve to tune the sensory afferent response in the cerebellar granular layer (Johansson and Birznicks, 2004). Finally, MF-GrC LTP in the cerebellar granular layer, together with other forms of long-term synaptic plasticity demonstrated in the molecular layer (Jörntell and Ekerot, 2002; Bing et al., 2015) and in deep cerebellar nuclei (Ohyama et al., 2006) under *in vivo* conditions, provides the spatiotemporal patterns of activity along the MF pathway, as well as an important determinant of cerebellar function.

## Data Availability Statement

The original contributions presented in the study are included in the article/supplementary material, further inquiries can be directed to the corresponding author/s.

## Ethics Statement

The experimental procedures were approved by the Animal Care and Use Committee of Yanbian University and were in accordance with the animal welfare guidelines of the U.S. National Institutes of Health. The permit number is SYXK (Ji) 2011-006.

## Author Contributions

D-LQ, C-PC, and Y-HB conceived and designed the experiments. DL, PW, and YL performed the experiments. C-PC, X-HJ, and D-LQ analyzed the data. C-PC and D-LQ wrote the manuscript. All authors contributed to the article and approved the submitted version.

## Conflict of Interest

The authors declare that the research was conducted in the absence of any commercial or financial relationships that could be construed as a potential conflict of interest.

## Publisher’s Note

All claims expressed in this article are solely those of the authors and do not necessarily represent those of their affiliated organizations, or those of the publisher, the editors and the reviewers. Any product that may be evaluated in this article, or claim that may be made by its manufacturer, is not guaranteed or endorsed by the publisher.
